# High-resolution mapping of a major and consensus quantitative trait locus for oil content to a ~ 0.8-Mb region on chromosome A08 in peanut (*Arachis hypogaea* L.)

**DOI:** 10.1007/s00122-019-03438-6

**Published:** 2019-09-26

**Authors:** Nian Liu, Jianbin Guo, Xiaojing Zhou, Bei Wu, Li Huang, Huaiyong Luo, Yuning Chen, Weigang Chen, Yong Lei, Yi Huang, Boshou Liao, Huifang Jiang

**Affiliations:** Key Laboratory of Biology and Genetic Improvement of Oil Crops, Ministry of Agriculture, Oil Crops Research Institute of the Chinese Academy of Agricultural Sciences, Wuhan, 430062 People’s Republic of China

## Abstract

**Key message:**

ddRAD-seq-based high-density genetic map comprising 2595 loci identified a major and consensus QTL with a linked marker in a 0.8-Mb physical interval for oil content in peanut.

**Abstract:**

Enhancing oil content is an important breeding objective in peanut. High-resolution mapping of quantitative trait loci (QTLs) with linked markers could facilitate marker-assisted selection in breeding for target traits. In the present study, a recombined inbred line population (Xuhua 13 × Zhonghua 6) was used to construct a genetic map based on double-digest restriction-site-associated DNA sequencing (ddRAD-seq). The resulting high-density genetic map contained 2595 loci, and spanned a length of 2465.62 cM, with an average distance of 0.95 cM/locus. Seven QTLs for oil content were identified on five linkage groups, including the major and stable QTL *qOCA08.1* on chromosome A08 with 10.14–27.19% phenotypic variation explained. The physical interval of *qOCA08.1* was further delimited to a ~ 0.8-Mb genomic region where two genes affecting oil synthesis had been annotated. The marker SNPOCA08 was developed targeting the SNP loci associated with oil content and validated in peanut cultivars with diverse oil contents. The major and stable QTL identified in the present study could be further dissected for gene discovery. Furthermore, the tightly linked marker for oil content would be useful in marker-assisted breeding in peanut.

**Electronic supplementary material:**

The online version of this article (10.1007/s00122-019-03438-6) contains supplementary material, which is available to authorized users.

## Introduction

Peanut (*Arachis hypogaea* L.) is one of the word’s major sources of vegetable oil. It is grown in more than 100 countries, with the global production of 43.98 Mt harvested from 27.66 Mha (FAOSTAT 2016). China, the world’s largest peanut producer, accounts for 38% of global peanut production. However, the domestic production of peanut hardly meets the increasing demands for peanut oil. Due to the large variation of oil content in the peanut germplasm (Yol et al. 2017), it is highly feasible to develop cultivars with high oil content and yield to enhance oil productivity. In addition, an increase of 1% in the oil content in peanuts could result in a 7% increase in economic benefit for oil processing (Shasidhar et al. 2017). Therefore, high oil content is a highly desirable trait which has been one of the key breeding objectives in many countries.

The trait of oil content is polygenetically inherited and is largely influenced by the environment (Baring et al. 2013; Wilson et al. 2013). Moreover, measuring oil content requires relatively expensive and complex equipment, and can be carried out only after harvest. Traditional breeding for improvement of oil content has a low efficiency in addition to being time-consuming. Marker-assisted selection (MAS) has provided a robust and reliable tool for accelerating the development of varieties with desirable target traits in many crops including peanut (Chu et al. 2011; Janila et al. 2016b; Varshney et al. 2014; Varshney 2016). However, the availability of well-validated genetic markers for the target trait is a prerequisite to deploy MAS in peanut. Previously, four minor quantitative trait loci (QTLs) were identified for oil content based on a genetic map with 45 SSR loci (Sarvamangala et al. 2011). Subsequently, six and nine QTLs for oil content were mapped on two genetic maps with 206 and 378 SSR loci, respectively (Pandey et al. 2014). Most recently, three QTLs for oil content have been identified in an advanced backcross population with 91 SSR markers (Wilson et al. 2017). All these studies could only develop low-density genetic maps leading to low-resolution of QTL mapping, while the genetic markers also lacked information on the physical location on the genome, which restricted further investigation and discovery of candidate genes and markers controlling oil accumulation.

Several efforts have been made previously for QTL discovery using sparse genetic maps constructed using SSRs (Huang et al. 2015; Varshney et al. 2009; Wang et al. 2012, 2015). Since the genetic base of cultivated peanut is narrow (Belamkar et al. 2011; Mukri et al. 2012), available SSR markers reported in earlier studies are limited for developing saturated genetic maps. However, single nucleotide polymorphisms (SNPs) are abundant and widely distributed across the genome. Based on the next-generation sequencing platform, double-digest restriction-site-associated DNA sequencing (ddRAD-seq) has been developed for SNP mining and genotyping in biparental mapping populations (Baird et al. 2008; Peterson et al. 2012). This method combines reduced representation libraries with multiplex sequencing strategies, which could overcome the complexity of the genome and reduce the cost of sequencing. Employing this technology has led to the successful development of high-density genetic maps (HDGMs) for conducting high-resolution mapping of QTLs in many crops (Chen et al. 2017; Chutimanitsakun et al. 2011; Liu et al. 2017; Pfender et al. 2011).

In peanut, few HDGMs have been deployed in QTL analysis for oil content. In the present study, ddRAD-seq was performed for the recombinant inbred line (RIL) population to address the following objectives: (1) construction of a HDGM based on SNPs, (2) identification of stable QTLs for oil content across multiple environments and delimitation of their physical regions in reference genomes, and (3) development of markers that are highly associated with oil content.

## Materials and methods

### Plant materials

The mapping population consisting of 186 RILs was derived from a cross between Xuhua 13 and Zhonghua 6 through the single seed descent method. The male parent (Zhonghua 6) is a Spanish-type variety belonging to *A. hypogaea *ssp. *fastigiata*. The female parent (Xuhua 13) is an intermediated-type variety, which has higher oil content than Zhonghua 6 (Table 1). Four generations of the RIL population, i.e., from F_5_ (2014) to F_8_ (2017) were used for phenotyping and the leaf samples from the F_6_ generation lines were used for DNA isolation and genotyping. In addition, a panel of 42 peanut cultivars with diverse oil content was collected from 14 provinces in China (Table S1) and phenotyping data in these cultivars were measured from 2014 to 2016.

**Table 1:** Description of phenotype analysis for oil content in the RIL population

Year	*P*1	*P*2	Range (%)	Mean (%)	SD	Kurt	Skew	*W* (Sig)
2014	51.52	47.06	43.19–51.8	47.83	1.67	− 0.08	− 0.10	1.00 (0.879)
2015	54.99	48.28	47.9–56.37	51.77	1.59	− 0.02	0.21	0.99 (0.351)
2016	53.34	49.35	45.30–54.20	50.09	1.60	− 0.29	− 0.09	0.99 (0.504)
2017	53.00	48.26	45.18–54.18	49.92	1.65	− 0.18	− 0.10	1.00 (0.937)

*P1* female parent; *P2* male parent *SD* standard deviation; *Kurt* kurtosis; *Skew* skewness; *W* Shariro–Wilk statistic value; *Sig* significance

### Field trials and phenotyping

The RIL population, their parents, and a panel of 42 cultivars were planted in the experimental field of OCRI-CAAS, Wuhan. Field trials were performed on a randomized complete block design with three replications. Each plot contained 12 plants at a spacing of 20 cm × 30 cm. Field management followed standard agricultural practice.

Both Oil% and H_2_O% in seeds were measured using nuclear magnetic resonance (PQ001; Niumag, China). Approximately 10 g seeds with less than 10% moisture content were weighed and analyzed for each of the three subsamples per entry. According to a previous study, oil content (%) was determined based on dry-weight using the formula {[oil%/(100 − H_2_O%)] × 100} (Pandey et al. 2014).

### Statistical analysis of phenotypic data

Phenotypic data were obtained from three biological replications in each field trial. The IBM SPSS Statistics software (v.22; IBM, USA) was used to analyze the phenotypic data. The normal distribution of phenotypic values was assessed by the Shapiro–Wilk test. Significant phenotypic differences among genotypes were assessed by Bonferroni’s Multiple Comparison Test. The field trial with three replications in each year was treated as a single environment, and the univariate variance analysis was performed to evaluate the effects of genotype, environment, and the interactions between genotype and environment on the phenotypic variance of oil content.

To estimate broad-sense heritability, the phenotypic variance was resolved into the genotypic variance ($\sigma_{\text{g}}^{2}$), the environmental variance ($\sigma_{\text{e}}^{2}$), the genotype × environment interaction variance ($\sigma_{{{\text{g}} \times {\text{e}}}}^{2}$), and the residual variance ($\sigma_{\varepsilon }^{2}$) using R (version 3.4.2) package “lme4” with the linear mixed model method (https://cran.r-project.org/web/packages/lme4/index.html). The entry mean-based broad-sense heritability of oil content trait was calculated as: $H^{2} = \sigma_{\text{g}}^{2} /(\sigma_{\text{g}}^{2} + \sigma_{{{\text{g}} \times {\text{e}}}}^{2} /r + \sigma_{\varepsilon }^{2} /rn)$, where r represents the number of environments and n represents the number of replications in each environment.

### Library construction and restriction-site-associated DNA (RAD) sequencing

Genomic DNA was extracted from young leaves of the RIL population and both parents using a genomic DNA extraction kit (TIANGEN, Beijing, China). Two endonucleases EcoRI (GAATTC) and MseI (TTAA), were applied to digest the genomic DNA. Library construction procedures have been previously described in detail (Baird et al. 2008; Wu et al. 2014). Finally, 250- to 450-bp fragments were excised and purified for pair-end sequencing. RAD sequencing was performed on a Hiseq4000 platform in BGI. Raw data have been deposited in GenBank (BioProject: PRJNA520741).

### SNP identification and genotyping

The software Stacks was used to identify SNPs and determine genotypes for the RIL population (Catchen et al. 2013). First, raw sequence reads were demultiplexed by their barcodes and low-quality reads were removed. Then, reads from each individual were clustered, aligned with each other, and scored for polymorphic loci. Putative loci markers with more than 50% missing data were filtered out. The remaining markers were mapped to two referenced genomes (*Arachis duranensis* and *A. ipaensis*) and only the uniquely mapped markers were used for the further construction of the genetic map.

### Genetic map construction and QTL analysis

The high-density linkage map was constructed using Joinmap software (Stam 1993). The mapping algorithm was based on regression mapping, and the Kosambi mapping function was applied to transform the recombinant ratio into genetic distance. Goodness of fit to the expected segregation ratio 1:1 for each locus was assessed by Pearson’s Chi square test (*P* < 0.01). SNP markers in the genetic map were aligned to chromosomes of the two diploid ancestors (*A. duranensis* and *A. ipaensis*) using the BLAST tool (https://blast.ncbi.nlm.nih.gov). CIRCOS software was applied to analyze the collinearity of the SNP loci between the genetic position and the physical position (Krzywinski et al. 2009).

The HDGM with multiple-years phenotypic data was used to identify QTLs for oil content. QTL IciMapping software, with inclusive composite interval mapping, was employed in the QTL analysis (Meng et al. 2015). The mapping parameters were set to 1.0 cM step, 0.001 probability, and a LOD threshold score of 2.5.

To compare current QTLs with previously reported QTLs, the physical positions of markers in the present and previous LGs, which harbored QTLs for oil content, were analyzed. After aligning the primer sequence of markers to the referenced genomes (*A. duranensis* and *A. ipaensis*), the markers in the current and earlier genetic maps were integrated into the corresponding chromosomes. The physical position of QTLs was estimated via linked markers.

### Annotation of genes in the QTL intervals

The markers linked to QTLs were aligned to the genomes of the two diploid ancestors of cultivated peanut (Bertioli et al. 2016). According to the physical position of flanking markers, annotated information on the putative genes in the QTL interval was downloaded from PeanutBase (https://www.peanutbase.org/home). Differentially expressed genes between high-oil cultivars and low-oil cultivars were obtained from Wang et al. (2018a).

To identify genes related to oil biosynthesis within the QTL interval, genes reported to be involved in oil biosynthesis in *Arabidopsis* were extracted from the proteome of *Arabidopsis* (Bates et al. 2014) and searched against the gene set in *A. duranensis* and *A. ipaensis* using the BLAST tool with an *E*-value cutoff of 1e−5 (https://blast.ncbi.nlm.nih.gov).

### Cluster analysis of gene expression profile

The software Genesis (v.1.7.6) was used to perform hierarchical clustering of genes within the seven QTLs that were identified in the current study (Sturn et al. 2002). Unweighted pair-group average linkage was used to calculate distances of gene clusters. The information on gene abundance in 22 tissues was downloaded from Clevenger et al. (2016). Genes with expression levels below 5 FPKM in any tissues were filtered out, and the remaining genes in the QTL intervals were used to make a heat map.

## Results

### Phenotypic analysis of oil content in RIL population

**Fig. 1:**
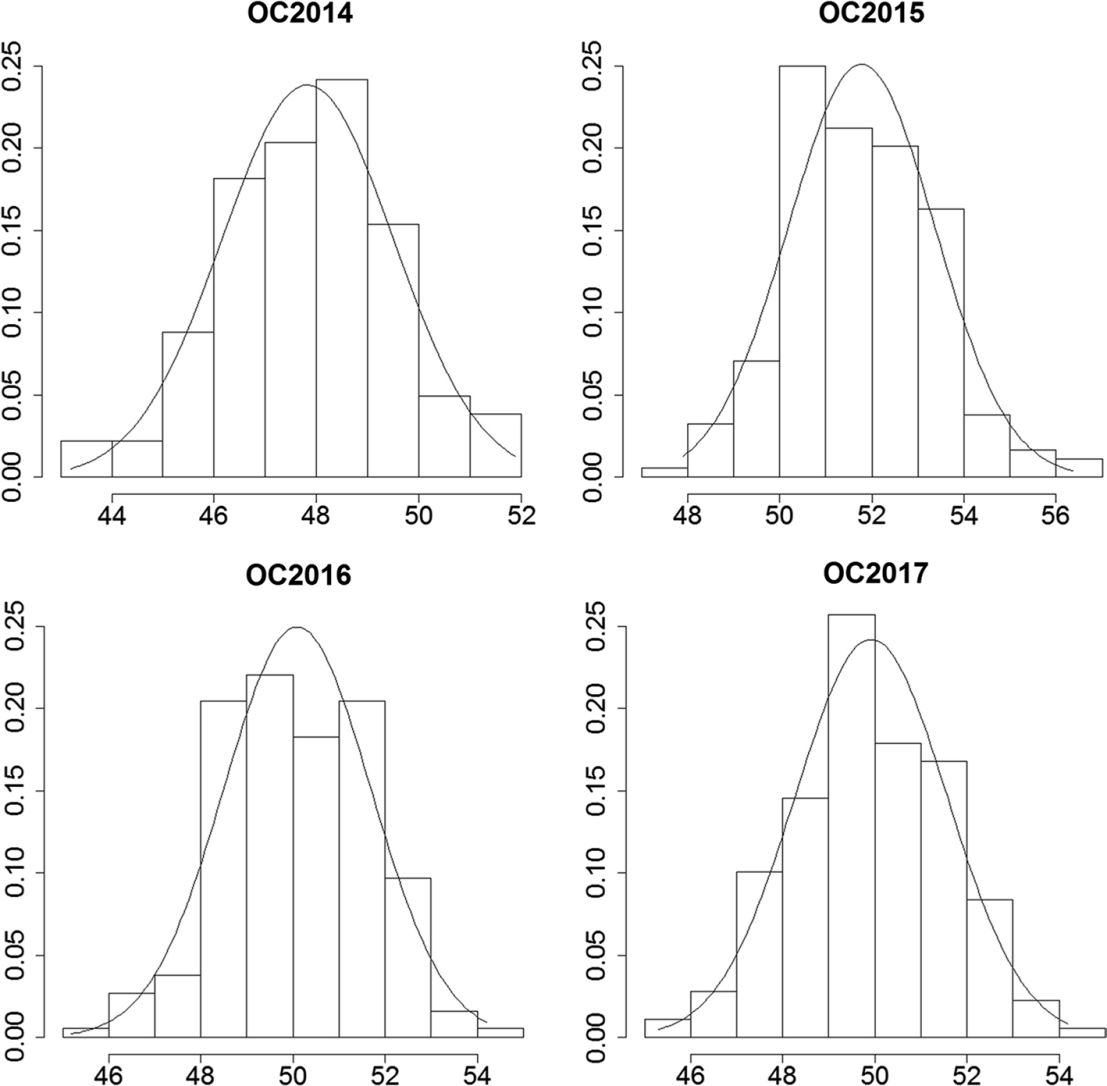
Phenotypic distribution of oil content in the RIL population across four consecutive years (2014–2017). The *y*-axis represents the density and the *x*-axis represents the oil content of seeds (%)

The oil content trait for 186 RILs and their parents were measured across four consecutive years (2014–2017). The oil content of the female parent (Xuhua 13) was consistently higher than that of the male parent (Zhonghua 6) across multiple environments (Table 1). The phenotypic data of the RIL population varied from 43.19 to 51.80% in 2014, from 47.90 to 56.37% in 2015, from 45.30 to 54.18% in 2016, and from 45.18 to 52.97% in 2017. Continuous distributions with transgressive segregation of phenotypic values of the RIL population are shown in Fig. 1. The Shapiro–Wilk (*w*) normality test indicates that the phenotypic data of the RIL population across four consecutive years were normally distributed (Table 1). Variance analysis across all four environments showed that genetic and environmental factors both significantly influenced oil content in RIL population at the *P* < 0.001 level (Table 2). However, interaction between genotype and environment only showed a moderate influence on the phenotype (*P* = 0.03). The broad sense heritability of oil content was estimated to be 0.84 in the RIL population.

**Table 2:** Analysis of variance for oil content across multiple environments

Source	*df*	Sum of square	Mean square	*F* value	*P* value
Genotype	186	2311.90	12.40	7.00	< 0.001
Environment	3	2131.30	710.40	400.06	< 0.001
Genotype × environment	549	1146.90	2.10	1.18	0.03
Error	524	930.50	1.80		

### Genotyping the RIL population through ddRAD-seq

After filtration of adapter and low-quality sequences, a total of 359.96 Gb of clean data (containing 3712.28 million reads) was obtained from RAD sequencing for 186 RILs and their parents. The Q20 ratio was 96.67% and the average GC content was 38.85% across the samples. High-quality reads of each individual were further aligned with each other and were subsequently clustered to RAD tags. The numbers of RAD tags in the female parent (Xuhua 13) and the male parent (Zhonghua 6) were 4.68 million and 4.65 million, respectively. Their corresponding sequencing depths were 55.33 and 53.13, respectively. In the RIL population, the RAD tag numbers ranged from 402,454 to 1,110,512 and the sequencing depths varied from 10.39 to 22.77 (Figs. S1A, S1B). These data were used to analyze SNPs and to catalog polymorphic loci. In total, 16,042 polymorphic loci across the whole RIL population were obtained. Considering that the two parents of the RIL population are homozygous, only aa × bb type loci containing 8064 SNPs markers were used in the construction of the genetic map. In each individual line, the number of genotyping aa × bb type loci ranged from 5377 to 7714 (Fig. S1C). These SNPs loci were further filtered using the stringent criterion described in “Materials and methods”, and 2595 SNP markers were mapped on the final genetic linkage map (Table S2).

### Construction of high-density genetic map

**Fig. 2:**
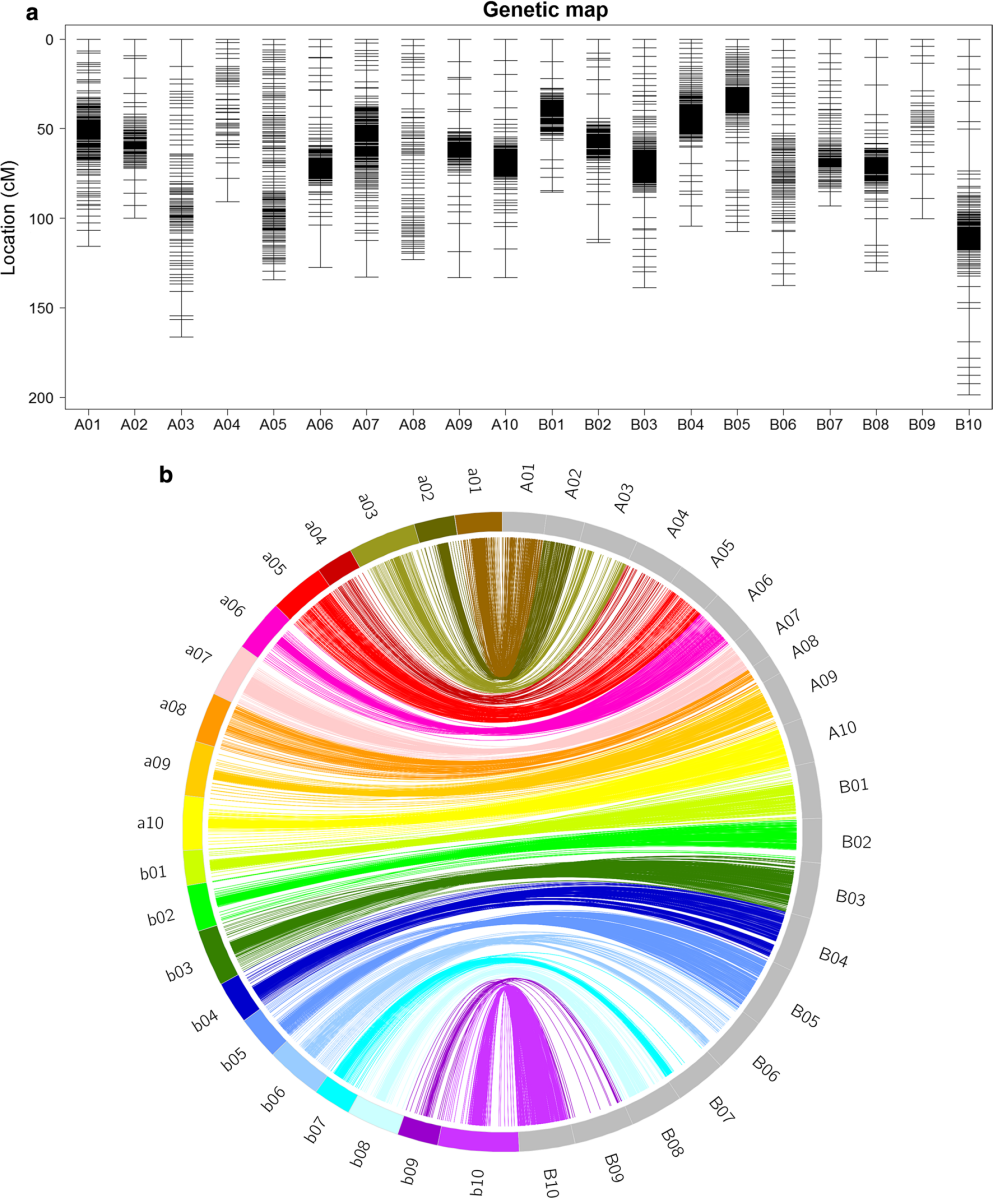
Overview of the genetic map constructed using SNP markers. **a** Distribution of SNP markers on 20 linkage groups. *Black bars* denote SNP markers. The linkage group numbers are shown on the *x*-*axis*. **b** Collinearity analysis between the genetic map and the physical map. *a01–a10* and *b01–b10* represent the 20 linkage groups. *A01–A10* and *B01–B10* indicate physical maps of *Arachis duranensis* and *Arachis ipaensis,* respectively

The final genetic map harbored 2595 SNP loci which covered a total distance of 2465.62 cM with an average length of 0.95 cM/locus (Fig. 2a; Table 3). LG A01–A10 were assigned to subgenome A, which contained 1235 loci with a total length of 1256.94 cM. LG B01–B10 were assigned to subgenome B, which contained 1360 loci spanning 1208.68 cM in length. Among the 20 LGs in the genetic map, 14 LGs have more than 100 loci. B10 was the largest LG, which harbored 218 loci with an average marker interval of 0.91 cM/locus. A04 and B09 harbored less than 50 loci and were the two shortest LGs in the genetic map. The average marker interval ranged from 0.51 cM/locus in B05 to 3.71 cM/locus in B09, and more than half of the LGs (12) had marker densities of below 1 cM/locus. The index of “Gap ≤ 5”, which was used to evaluate the degree of linkage of between markers, was shown to have an average value of 96.11%. The largest gaps in the genetic map were 23.54 cM on LG A06, followed by 23.43 cM on LG B10. Among the 2595 loci in the genetic map, 18.6% of loci (483) were shown to have distorted segregation (*P* < 0.01). LG B05 harbored the largest number of distorted loci (152), whereas LG A08, B09, and B10 contained less than four distorted loci.

**Table 3:** Summary of the high-density genetic map for the RIL population

Linkage group	Loci	Total length (cM)	Interval (cM/loci)	Largest gap (cM)	Gaps > 5^a^ (%)	SDL^b^
A01	169	115.80	0.69	9.01	1.78	15
A02	101	100.00	0.99	10.91	6.93	14
A03	93	166.38	1.79	15.07	4.30	5
A04	47	90.71	1.93	13.03	6.38	12
A05	145	134.22	0.93	4.51	0.00	77
A06	139	127.47	0.92	23.54	3.60	42
A07	175	132.78	0.76	20.33	1.14	12
A08	69	123.04	1.78	8.64	4.35	2
A09	137	133.28	0.97	15.76	6.57	48
A10	160	133.25	0.83	16.05	5.00	9
A subgenome	1235	1256.94	1.02	–	3.48	236
B01	120	85.33	0.71	12.47	5.00	12
B02	130	113.48	0.87	19.44	4.62	6
B03	186	138.69	0.76	11.30	3.23	7
B04	169	104.45	0.62	11.31	2.96	14
B05	212	107.38	0.51	10.78	1.42	152
B06	93	137.52	1.48	12.04	7.53	11
B07	84	93.08	1.11	8.01	4.76	33
B08	121	129.73	1.07	16.67	4.13	9
B09	27	100.28	3.71	15.32	18.52	0
B10	218	198.74	0.91	23.43	5.05	3
B subgenome	1360	1208.68	0.89	–	4.27	247
Total	2595	2465.62	0.95	–	3.89	483

^a^*Gaps > 5 *indicates the percentage of gaps in which the distance between adjacent markers was larger than 5 Cm

^b^*SDL* denotes the number and percentage (within the parenthesis) of segregation distortion loci in each linkage group (*P* < 0.01)

To further assess the quality of the genetic map, collinearity analysis was conducted to compare between the genetic and physical positions of the SNP loci. Each locus in the genetic map was mapped to the referenced genomes. The physical distance between the start and end loci of each LG were estimated to cover 80% of the total genomes of the two diploid ancestors. Most loci in the genetic map had the same order as those on the corresponding chromosomes of the two reference genome generally. LGs assigned to subgenome A had better compatibility with the physical map than LGs assigned to subgenome B (Fig. 2b). LGs A03, B06, B07 and B09 showed a degree of deviation in the collinearity analysis.

### Identification of quantitative loci for oil content trait

In total, seven QTLs for oil content were mapped to the HDGM across four environments (Fig. S2; Table 4). The seven QTLs with 6.07–27.19% explained phenotypic variation (PVE), located on five LGs, namely, LG A04 (*qOCA04.1*), LG A05 (*qOCA05.1*), LG A08 (*qOCA08.1* and *qOCA08.2*), LG B05 (*qOCB05.1* and *qOCB05.2*) and LG B06 (*qOCB06.1*) (Table 4). The genetic distance of the QTL intervals ranged from 0.8 cM for *qOCB06.1* to 7.1 cM for *qOCB05.2*, and the average distance of QTL interval was below 2.6 cM. Based on the sequences of flanking markers, these QTLs were mapped to the reference genomes of *A. duranensis* and *A. ipaensis*. The physical intervals of QTLs ranged from 0.3 Mb to 2.5 Mb, and the number of genes within these intervals varied from 12 to 217 (Tables 4, S3). When BLAST was performed with these genes against homologous genes in *Arabidopsis* (Bates et al. 2014), a total of 22 putative genes were found to be involved in 11 oil-related pathways. These pathways included plastidial glycerolipid, galactolipid and sulfolipid synthesis, sphingolipid synthesis, aromatic suberin synthesis, lipid-related synthesis, lipid signaling, eukaryotic phospholipid synthesis, cuticular wax synthesis, lipase synthesis, cutin synthesis, triacylglycerol biosynthesis and triacylglycerol degradation. In addition, 21 putative transcription factor genes belonged to 15 families, specifically FAR1, bHLH, MYB-related, G2-like, WRKY, TALE, NF-Y, AP2, HSF, BES1, HD-ZIP, bZIP, C2H2, MYB, and NAC (Wilson et al. 2008). Based on publicly released transcriptome data for tetraploid peanut (*Arachis hypogaea* cv. Tifrunner, Clevenger et al. 2016), genes abundances in 22 tissues were obtained including complete seed developmental stages. Genes in the seven QTL intervals could be classified into four subgroups, i.e., Sub A, B, C and D, based on their expression profiles (Fig. S3, Table S3). In Sub A, most genes were highly expressed in leaves. In Sub B, most genes were highly expressed in shoot tips. In Sub C, the expression levels of most genes were relatively higher in roots and reproductive tissues than in leaves, flowers, and pistils. In Sub D, more than half of the genes were predominantly expressed in seeds at different developmental stages. In addition, 36 genes in the QTL intervals have been reported to be differentially expressed between high-oil cultivars and low-oil cultivars (Wang et al. 2018a). Of these 36 genes, three oil-related genes and one transcription factor gene were upregulated in high-oil cultivars (Table S3).

**Table 4:** QTL analysis for oil content trait in four environments

QTL	LG	Environment	Marker interval	Additive effect	LOD	PVE (%)	Length	Gene number
*qOCA04.1*	A04	2016	AhMXZ28711–AhMXZ257725	− 0.41	3.31	6.51	1.7 cM (2.5 Mb)	217
*qOCA05.1*	A05	2017	AhEXZ406582–AhEXZ421090	− 0.47	3.79	7.32	2.0 cM (0.6 Mb)	42
*qOCA08.1*	A08	2014	AhMXZ190701–AhEXZ283046	0.59	4.83	10.14	3.7 cM (0.8 Mb)	49
		2016		0.83	14.35	27.19		
		2017		0.65	6.53	13.89		
*qOCA08.2*	A08	2015	AhEXZ21087–AhEXZ259735	0.70	9.83	19.20	7.1 cM (0.3 Mb)	12
*qOCB05.1*	B05	2016	AhEXZ407027–AhMXZ297422	− 0.42	4.03	6.91	1.6 cM (0.9 Mb)	54
*qOCB05.2*	B05	2015	AhEXZ248401–AhEXZ172745	− 0.41	3.32	6.39	1.0 cM (0.8 Mb)	50
*qOCB06.1*	B06	2017	AhEXZ86078–AhEXZ351422	0.43	2.82	6.07	0.8 cM (2.1 Mb)	95

*PVE* phenotypic variation explained; *Length* genetic and physical (within the parentheses) distance of marker interval; *LOD* logarithm of odds

**Fig. 3:**
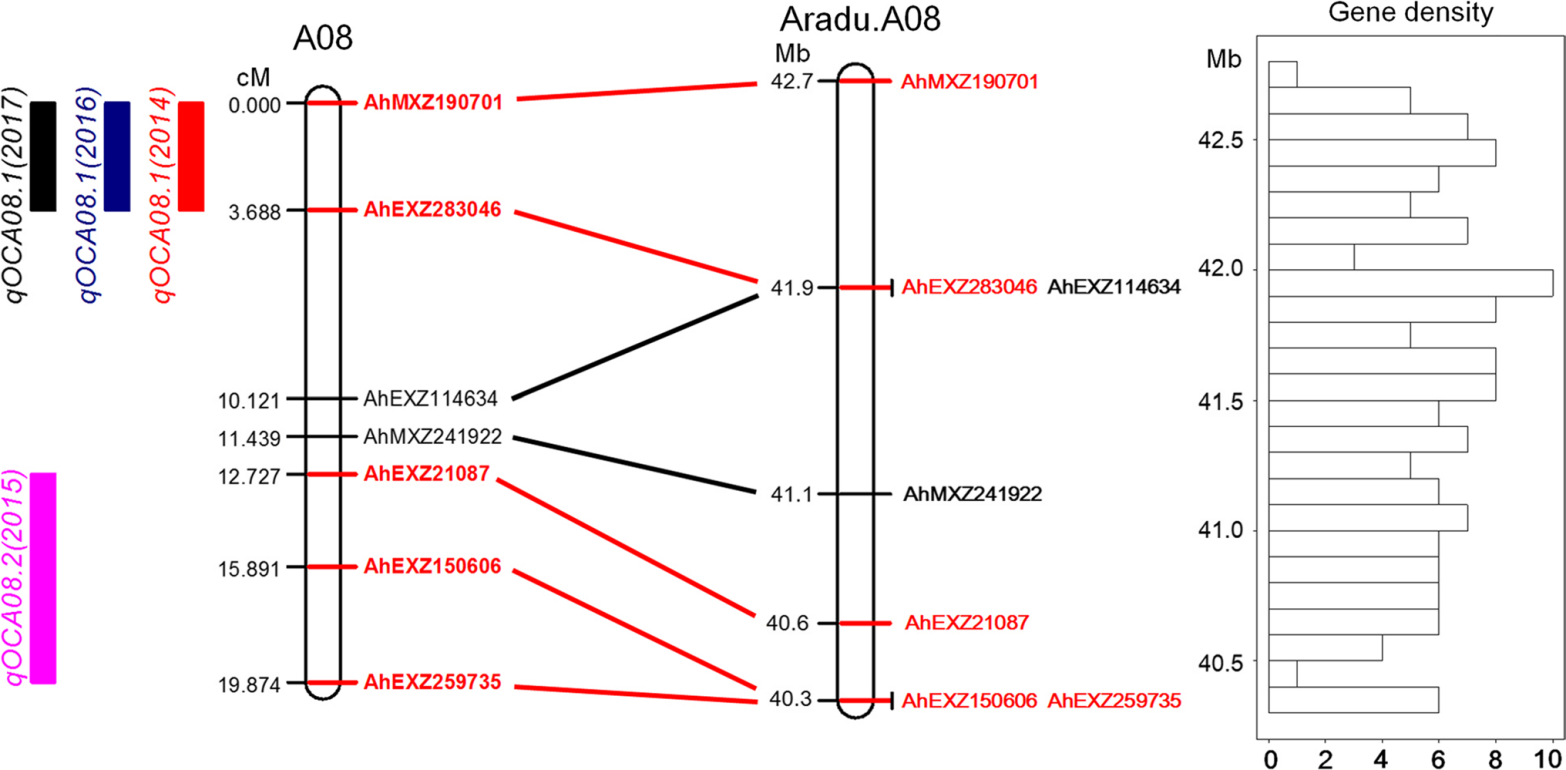
Location of QTLs on LG A08 and the corresponding physical map. The *different colored boxes* represent the QTLs detected during different years. The markers that tightly link to the QTL are highlighted in *red*. Gene density represents gene number per 80-kb interval

Among the seven QTLs, *qOCA08.1* with 10.14–27.19% PVE could be repeatedly detected across 3 years. The consensus QTL *qOCA08.1* between the markers AhMXZ190701 and AhEXZ283046 was aligned to a ~ 0.8-Mb (41.9–42.7 Mb) genomic region of pseudomolecule A08 of *A. duranensis* (Fig. 3). After resequencing the genomes of the two parents (Appendix S1), 38 SNPs were found to be within the physical region of *qOCA08.1* (Table S4). Two SNPs were located on the introns of two genes that encoded a calcium-dependent protein, Annexin (*Aradu.N8MUP*), and a flavin-binding monooxygenase family protein (*Aradu.1F2UP*). Six SNPs were located upstream or downstream of six genes that encoded the proline-rich family protein (*Aradu.VWM5Q*), Ulp1 protease family proteins (*Aradu.VZ1DU* and *Aradu.D7VM0*), NF-Y transcription factor (*Aradu.S0YHI*), unknown proteins (*Aradu.F5MYE*), and flavin-binding monooxygenase family protein (*Aradu.1F2UP*). The remaining 30 SNPs were located in intergenic regions.

### Development and validation of markers for oil content

**Fig. 4:**
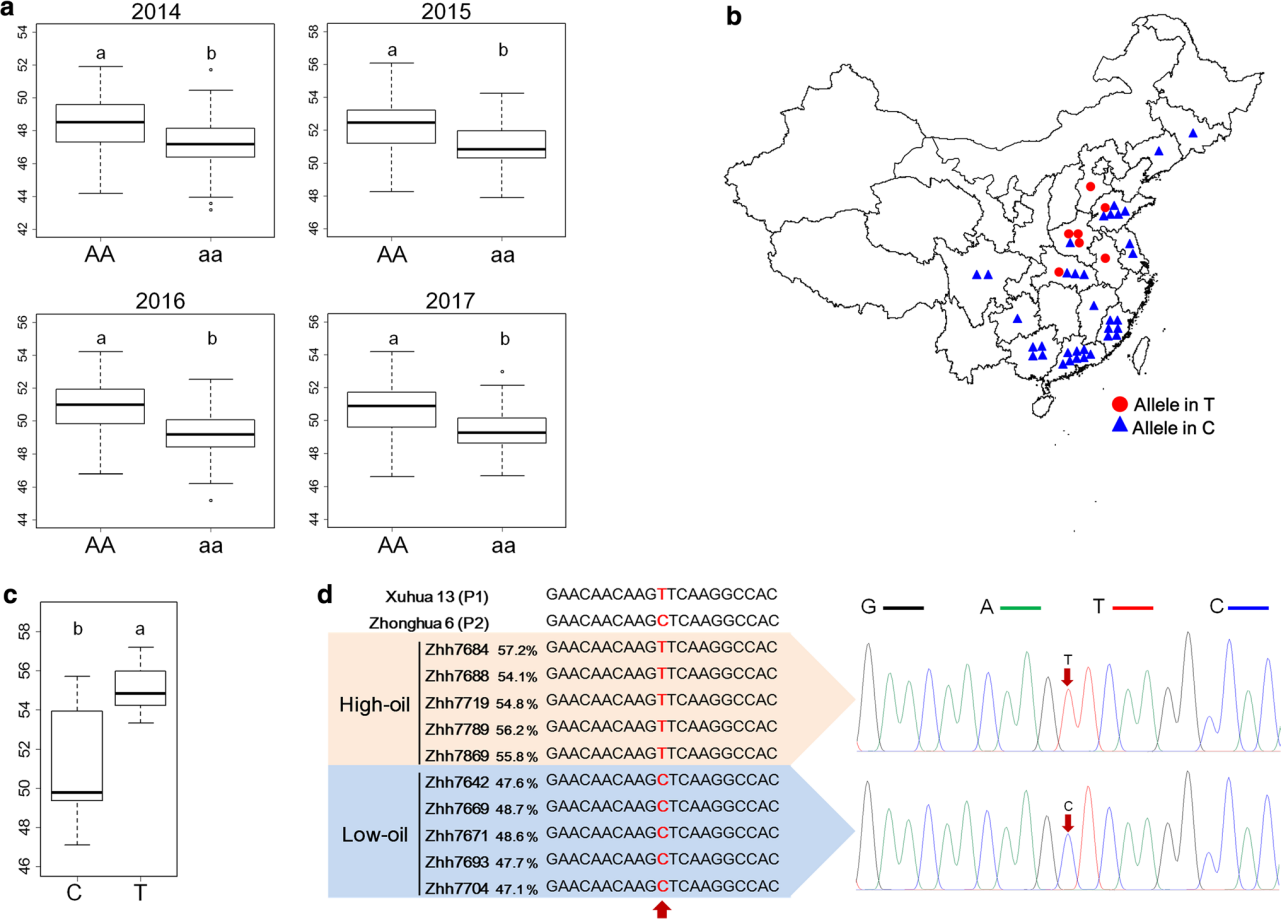
Validation of the *AhMXZ190701* SNP locus for high-oil content selection**. a** Phenotypic difference between two genotypes at the *AhMXZ190701* SNP locus in the RIL population. The AA homozygous allele originated from Xuhua 13 (female parent) and the aa homozygous allele originated from Zhonghua 6 (male parent). **b** Geographic distribution of the 42 cultivars in China. Cultivars carrying nucleotide T at the SNP site are represented by a *red circle*. Cultivars carrying nucleotide C at the SNP site are denoted by a *blue triangle*. **c** Phenotypic difference between accessions with nucleotide C and accessions with nucleotide T at the AhMXZ190701 site. **d** Validation of SNP-based markers in high-oil and low-oil cultivars and in the parents of RIL population (Xuhua 13 and Zhonghua 6). Within plots, the *bars with different letters* are significantly different according to Bonferroni’s Multiple Comparison Test (*P* < 0.001)

The flanking markers of QTL *qOCA08.1* were AhMXZ190701 and AhEXZ283046. For the *AhMXZ190701* locus, oil contents of the lines that harbored the homozygous allele of the female parent were significantly higher than that of the male parent across four consecutive years (*P* < 0.01; Fig. 4a; Table S5). Similarly, a significant difference in oil content was found between lines with alleles from the female parent and the male parent at the *AhEXZ283046* locus (Table S5). Values for agronomic traits, 100-pod weight, and plant height of lines with alleles from the male parent did not significantly differ from lines with alleles from the female parent at two loci (AhMXZ190701 and AhEXZ283046) (*P* < 0.05; Table S6). Since QTL *qOCA08.1* had a large effect on oil content across multiple environments, the tightly linked SNP markers, AhMXZ190701 and AhEXZ283046, are a potentially valuable application in MAS for a high-oil trait.

Both SNP sites were then validated in a panel of 42 cultivars collected from 14 provinces across China (Fig. 4b; Table S1). The oil contents of these 42 cultivars ranged from 47.09 to 57.19% on average over a 3-year period (2014–2016), with mean of 51.61% (Table S1). The resequencing of the genomes of these 42 cultivars (Appendix S1) indicated that the AhMXZ190701 site was polymorphic. Seven accessions had the nucleotide T at the AhMXZ190701 site, while the remaining accessions had the nucleotide C at this site (Fig. 4b; able S1). Of accessions with nucleotide T at the AhMXZ190701 site, the average oil content exceeded 54.9%, whereas the oil content of accessions with nucleotide C at this site was significantly lower, with an average of only 50.9% (*P* < 0.01; Fig. 4c). It is worth noting that nucleotide T at the AhMXZ190701 site did not occur in any low-oil accessions (oil content < 50%). These results suggested that the A*hMXZ190701* SNP locus was highly associated with the trait of oil content and may be a suitable diagnostic marker candidate. Therefore, the marker SNPOCA08 was developed to amplify 561-bp fragments, containing the AhMXZ190701 site (Table S7), from five high-oil cultivars (Zhh7684, 7719, 7789, 7689, and 7688), five low-oil cultivars (Zhh7704, 7642, 7693, 7669, 7671), and the two parents of the RIL population (Xuhua 13 and Zhonghua 6). Sanger sequencing showed that this marker could distinguish between high-oil varieties with 54.1–57.2% oil content and low-oil varieties with 47.1–48.6% oil content, based on nucleotide variation at the AhMXZ190701 site. These results demonstrated that the marker SNPOCA08 could be useful for selecting high-oil lines in the peanut breeding process.

## Discussion

Improving oil content of seeds is an important objective for peanut breeding. MAS is an effective tool for accelerating the breeding process, which has been widely deployed in the development of varieties with disease resistance and desirable fatty acid composition in peanut (Chu et al. 2011; Janila et al. 2016a). Employment of MAS for high-oil breeding requires identification of QTLs with closely linked makers for oil content. However, the allotetraploid peanut has a narrow genetic base, which hinders development of a dense genetic map for QTL fine mapping. Based on the next generation sequencing platform, ddRAD-seq could be applied for large-scale SNP mining and genotyping. It is an effective and relatively inexpensive method to construct HDGM. Hence, it has been widely used in many crops for QTL mapping (Baird et al. 2008; Chen et al. 2017; Pei et al. 2018; Zhang et al. 2016; Zhou et al. 2018). In 2014, the first HDGM was constructed for allotetraploid peanut through ddRAD-seq. Subsequently, based on SNP markers, a number of HDGMs were applied for the QTL identification of several agronomic traits, such as fatty acid composition and disease resistance (Agarwal et al. 2018; Hu et al. 2018; Vishwakarma et al. 2016; Wang et al. 2018b; Zhou et al. 2014). In the present study, we constructed and deployed an HDGM in QTL identification and marker development for oil content in peanut. This genetic map consisted of 2595 loci, spanning 2465.62 cM with an average distance of 0.95 cM/locus (Table 3). To our knowledge, the number of independent genetic loci in this map approaches or even exceeds that of other recently reported HDGMs for peanut (Agarwal et al. 2018; Hu et al. 2018; Wang et al. 2018b). Dependent on the same RIL population, a genetic map, consisting of 817 SSR markers, has been developed with a marker interval of 2.1 cM/locus (Luo et al. 2017). The density of the genetic map in the present study has been considerably improved, which could provide more tightly linked markers. In addition, each SNP locus of the HDGM in this study could be precisely mapped to the reference genomes of diploid progenitors, which enables deeper insight into the target genomic location. In the current study, the physical length of QTL intervals ranged from 0.3 to 2.5 Mb, which was identified by locating flanking SNP markers on reference genomes of *A. duranensis* and *A. ipaensis* (Table 4). The average number of genes in the QTL intervals was below 75, and lowest number was only 12, for *qOCA08.2* (Table S3).

A total of seven QTLs on five LGs were identified in the current study (Table 4). Previous studies have also observed that the trait of oil content was controlled by multiple QTLs, indicating that oil content is polygenetically inherited (Pandey et al. 2014; Shasidhar et al. 2017; Wang et al. 2018a; Wilson et al. 2017). According to the physical position of linked markers in the reference genomes of the two diploids, seven QTLs in the present study were mapped to pseudomolecules A04, A05, and A08 in *A. duranensis*, and to pseudomolecules B05 and B04 in *A. ipaensis*. Previously reported QTLs with linked markers for oil content were also located on A04, A05, and A08 (Fig. S4; Pandey et al. 2014; Sarvamangala et al. 2011; Shasidhar et al. 2017; Wilson et al. 2017). For instance, marker PMc348 has been reported to be linked to QTL for oil content and was mapped to 82.3 Mb of A04 (Wilson et al. 2017). However, in the present study, *qOCA04.1* was located on a 3.6- to 6.1-Mb interval of A04. Two additional and previously identified QTLs for oil content were found to be located within the marker interval of GM1878 and GM1890, and the marker interval of TC2b09 and RN16F05, which were mapped to the 6.4- to 10.9-Mb and 15.0- and 21.7-Mb regions of A05, respectively (Pandey et al. 2014; Sarvamangala et al. 2011). In the current study, *qOCA05.1* was located on the 102.0- to 102.6-Mb regions of A05. It is our understanding that the two parents of the current RIL population (Xuhua 13 × Zhonghua 6) did not have a close genetic relationship with populations previously evaluated for oil content, such as RIL from SunOleic 97R × NC94022, RIL from TG 26 × GPBD 4, and BC_3_F_6_ from Florunner × TxAG-6. Thus, the *qOCA04.1* and *qOCA05.1* in the present study seem not to be the same as previously described QTLs on A04 and A05. However, using linked markers sometimes did not accurately estimate the physical position of the QTLs. The discrepancies in positions between current and previous QTLs may also be due to the relatively low density of previous genetic maps. On chromosome A08, markers Seq 2A05 (Alias 2A5) and TC1E05, that have been previously reported, were found to be adjacent to *qOCA08.1* and *qOCA08.2* on A08 (Fig. S4). According to the flanking markers, *qOCA08.1* was mapped to the 41.9- to 42.7-Mb position of A08. The QTL *qOCA08.2* was located approximately 1.3 Mb upstream of *qOCA08.1* (Fig. 3). Among the different accessions of *A. duranensis*, 2A5 was a polymorphic marker and was found to be highly correlated with the oil content trait (Huang et al. 2012). This marker was located at the 39.9-Mb position on chromosome A08, which is close to *qOCA08.1* and *qOCA08.2*. TC1E05 was a flanked marker of *mqOC* for oil content which was detected in the RIL population from SunOleic 97R × NC94022 (Pandey et al. 2014). This marker was mapped to ~ 1.1 Mb downstream of QTL *qOCA08.1* (~ 43.8 Mb of A08). Although it cannot yet be confirmed that *qOCA08.1* and *qOCA08.2* are the same as previously detected QTLs based on the flanking markers, it is reasonable to assume that the physical region of 39.9–43.8 Mb on chromosome A08 is a hot spot for genes controlling oil content.

The QTL *qOCA08.1* with 10.14–27.19% PVE in this region was repeatedly detected in multiple environments, which indicated that it may be a valuable candidate for further fine mapping. In the 0.8-Mb physical region of *qOCA08.1*, eight SNPs between two parents were found to be located either upstream or downstream, or as an intron of seven genes (Table S4). Of these, *Aradu.S0YHI* was predominantly expressed in a series of reproductive tissues, in which triacylglycerol was synthesized and accumulated (Fig. S3; Table S3). *Aradu.S0YHI* encoded a transcription factor that belongs to the NF-Y family. Several members of the NF-Y transcription factor family (e.g., *LEC1*, *ABI3*, and *FUS3*) have been shown to play important roles in the regulation of oil accumulation in seeds (Lu et al. 2016; Mu et al. 2008; Tang et al. 2018). One SNP was detected 38 bp upstream of the start codon for *Aradu.S0YHI*. However, the link between this variant and gene function remains unclear. In addition to *Aradu.S0YHI*, *Aradu.1F2UP* has been reported to be differentially expressed between high-oil cultivars and low-oil cultivars (Wang et al. 2018a). *Aradu.1F2UP* encodes a flavin-binding monooxygenase family protein, which plays a key role in auxin biosynthesis (Yi et al. 2013). Auxin exerts significant effects on plant development and metabolism, which includes oil accumulation (Zhao 2010, Jusoh et al. 2015). Two SNPs were found to be located 72 bp upstream of the intron of *Aradu.1F2UP*. It will be interesting to further investigate whether the variant in the promoter of *Aradu.1F2UP* would lead to a difference in the transcript abundance between both parents of the RIL population. Although SNP analysis, expression profile analysis, and gene annotation indicate these putative genes in the QTL interval as candidates for controlling oil content, it is still possible that key genes may sometimes exist outside the QTL interval between two significant markers. Further studies that include transgenic assays should be performed in the future to verify these putative genes for oil content.

In the present study, the SNP marker AhMXZ190701 was tightly linked to the major and stable QTL *qOCA08.1*. Whether in the RIL population or in a panel of cultivars, there was a significant difference in oil content between the allelotypes with nucleotide T and allelotypes with nucleotide C at site AhMXZ190701 (Fig. 4; Table S5). Agronomic traits for the RIL population, such as 100-pod weight and plant height, have been previously assessed (Lv et al. 2018; Luo et al. 2017). Phenotypic differences for these additional agronomic traits across multiple environments were not observed among different genotypes of the SNP marker AhMXZ190701 (Table S6). These results suggested that the SNP *AhMXZ190701* locus may be highly associated with the oil content trait, while it may not be linked with additional agronomic traits. Based on the *AhMXZ190701* locus, the marker SNPOCA08 was developed to distinguish high-oil and low-oil cultivars through Sanger sequencing (Fig. 4d). Since the oil content trait is affected by the environment, the deployment of the marker SNPOCA08 for trait selection can help to enhance the efficiency of high-oil breeding. It is notable that most peanut cultivars (35 of 42) selected from the main production areas in China did not have the high-oil allele (nucleotide A) at the *AhMXZ190701* locus. Consequently, this marker may be valuable for introducing the superior allele into peanut cultivars for improvement of oil content.

In summary, we constructed a high dense genetic map covering 2595 loci with an average distance of 0.95 cM/locus. Using 4-year phenotypic data of the RIL population, seven QTLs for the oil content were identified by high-resolution mapping. The consensus QTL *qOCA08.1* with up to 27.19% PVE was located in a 0.8-Mb interval of chromosome A08, which contained two genes influencing oil synthesis. This stable and major QTL allowed us to develop a marker SNPOCA08 for the flanked SNP site AhMXZ19070 associated with the trait of oil content. These results will facilitate the further investigation of the genetic basis of oil synthesis, and may also provide a useful marker in MAS for a high-oil trait in peanut.

## Electronic supplementary material

Below is the link to the electronic supplementary material.

Supplementary material 1 (DOCX 1133 kb)

Supplementary material 2 (XLSX 386 kb)
