# Supplementary material for: High-resolution mapping of a major and consensus quantitative trait locus for oil content to a ~ 0.8-Mb region on chromosome A08 in peanut (Arachis hypogaea L.)
Source: Theor Appl Genet. 2019 Sep 26;133(1):37–49. doi: 10.1007/s00122-019-03438-6 (PMC6952344; doi:10.1007/s00122-019-03438-6)
Supplement: Supplementary file 1 — Supplementary material 1 (DOCX 1133 kb) [file 122_2019_3438_MOESM1_ESM.docx]

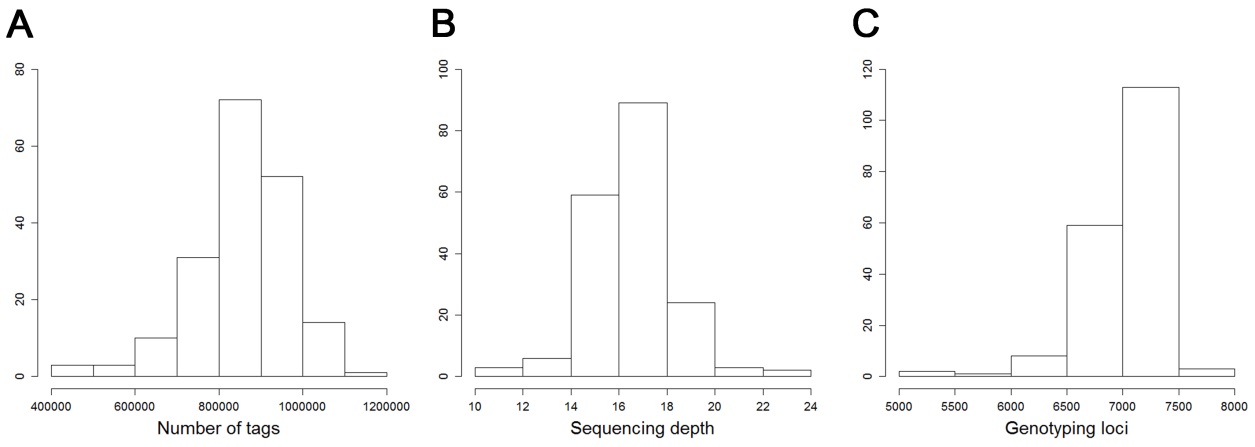


**Fig. S1** Detailed information on sequencing data in the RIL population. (A) Distribution of the number of tags in the RIL population. (B) Distribution of average loci of sequencing depth in the RIL population. (C) Distribution of the number of genotyping loci in the RIL population.


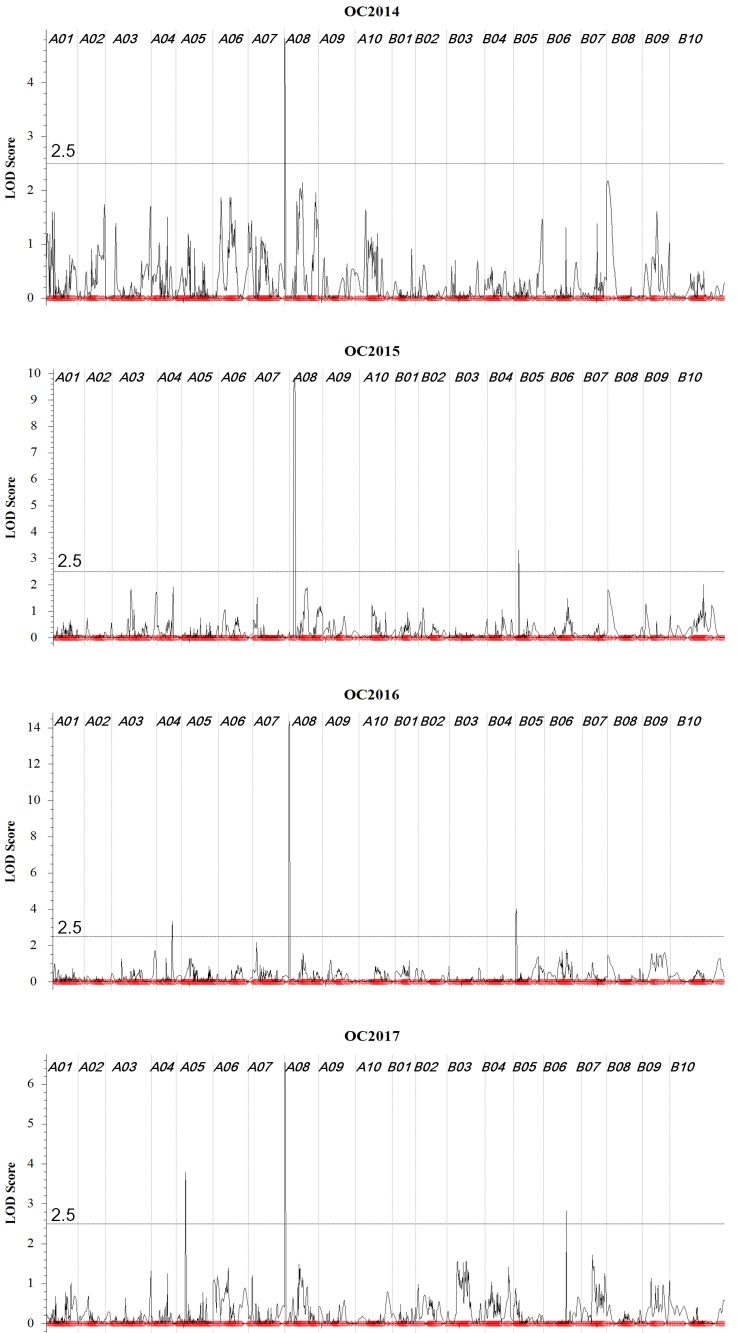


**Fig. S2** Genome-wide overview of QTLs for oil content across four consecutive years (2014-2017).


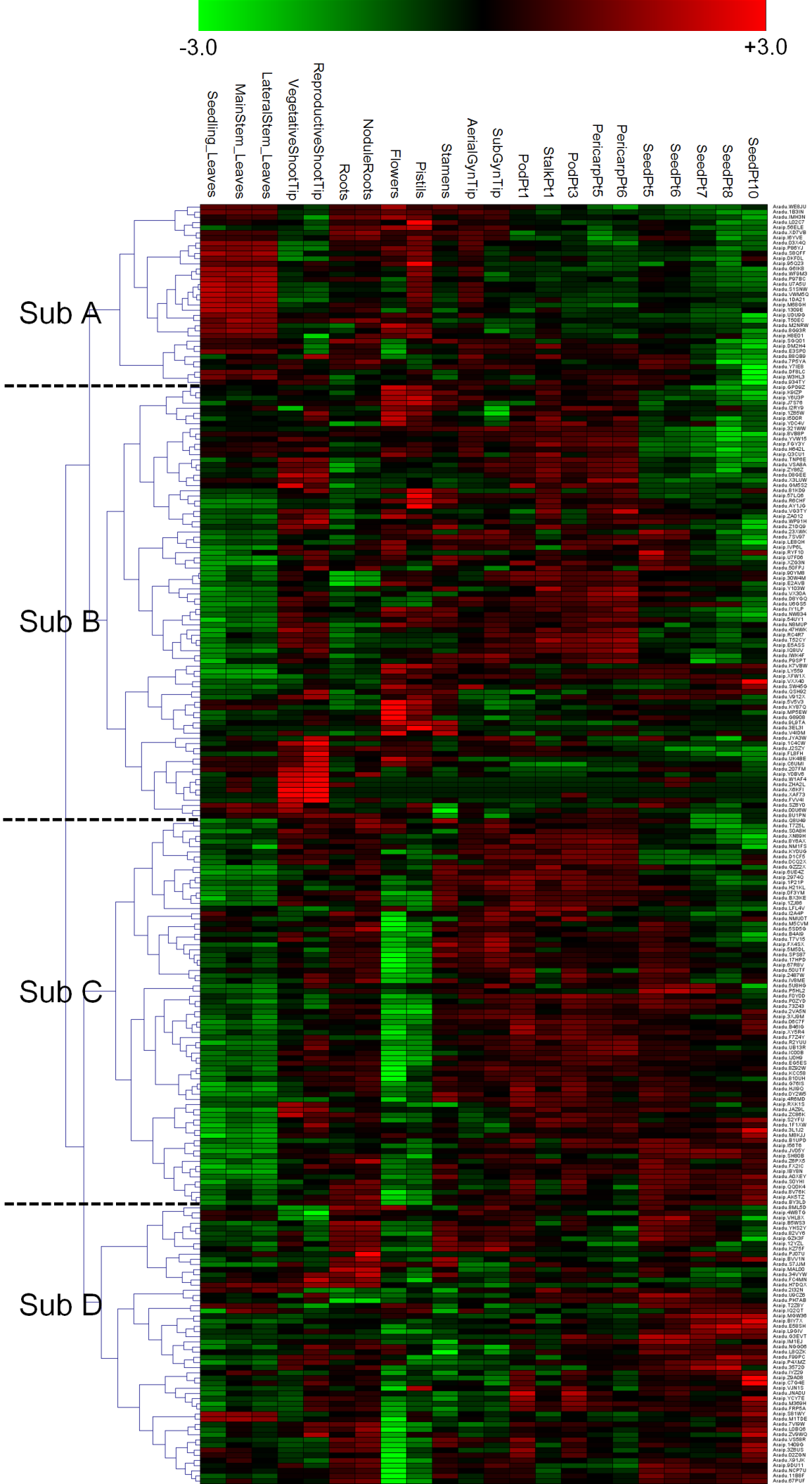


**Fig. S3** Expression pattern of genes in 22 peanut tissues. Genes are clustered into four subgroups (Sub A, B, C, and D) based on their expression levels in 22 tissues.


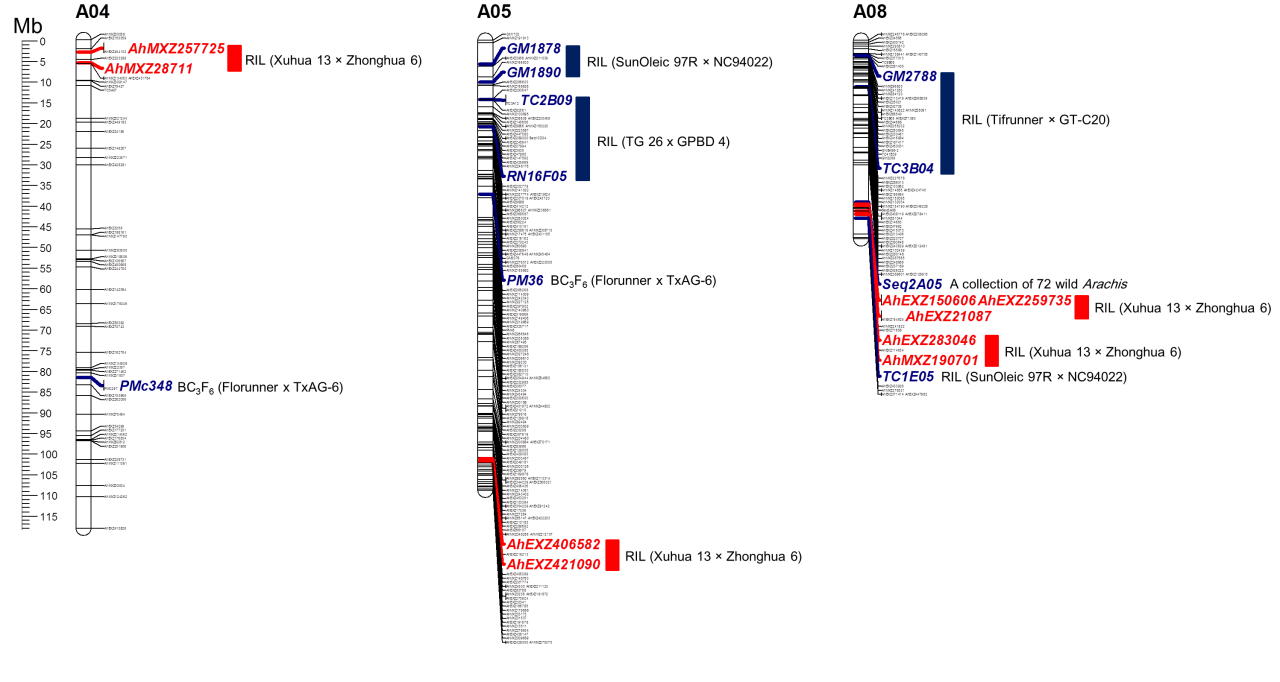


**Fig. S4** Integration of the currently and previously identified QTLs into chromosomes of *A. duranensis*. Markers in blue indicate that they were previously reported to be associated with oil content. Markers in red color indicate that they were detected to be highly linked with QTLs for oil content in the present study. Blue rectangles and red rectangles denote the QTLs identified in previous studies and present study, respectively.

**Appendix S1**

**Genome re-sequencing**

Two parents of the RIL population and 42 cultivars from China have been sampled for genome re-sequencing. High-quality genomic DNA from a single plant of each accession was extracted using a Plant Genomic DNA Kit (TIANKEN, Being, China). About 400-bp paired-end (PE) libraries were constructed using the Illumina TruSeq DNA sample prep kit. Sequencing was performed on the Illumina HiSeq platform. Raw data were trimmed to remove low quality bases, reads with more than 2% unidentified nucleotides, and adaptors, using Trimmomatic (Bolger et al. 2014). Clean reads were aligned to two diploid genomes using BWA software (Li and Durbin 2009). BAM alignment files were subsequently generated with SAMtools (Li et al. 2009) and were used for SNP calling among accessions by the software GATK (McKenna et al. 2010).

**Reference**

Bolger AM, Lohse M, Usadel B (2014) Trimmomatic: a flexible trimmer for Illumina sequence data. Bioinformatics 30: 2114-2120

Huang L, Jiang HF, Ren XP, Chen YN, Xiao YJ, Zhao XY, Tang M, Huang JQ, Upadhyaya HD, Liao BS (2012) Abundant microsatellite diversity and oil content in wild *Arachis* species. PLoS ONE 7: e50002

Li H, Durbin R (2009) Fast and accurate short read alignment with Burrows-Wheeler transform. Bioinformatics 25: 1754-1760

Li H, Handsaker B, Wysoker A, Fennell T, Ruan J, Homer N, Marth G, Abecasis G, Durbin R, and 1000 Genome Project Data Processing Subgroup (2009) The Sequence alignment/map (SAM) format and SAMtools. Bioinformatics 25: 2078-2079

Lv J, Liu N, Guo J, Xu Z, Li X, Li Z, Luo H, Ren X, Huang L, Zhou X, Chen Y, Chen W, Lei Y, Tu J, Jiang H, Liao B (2018) Stable QTLs for plant height on chromosome A09 identified from two mapping populations in peanut (Arachis hypogaea L.). Front Plant Sci 9: 684

Luo H, Guo J, Ren X, Chen W, Huang L, Zhou X, Chen Y, Liu N, Xiong F, Lei Y, Liao B, Jiang H (2017) Chromosomes A07 and A05 associated with stable and major QTLs for pod weight and size in cultivated peanut (Arachis hypogaea L.). Theor Appl Genet 131: 1-16

Mckenna A, Hanna M, Banks E et al. (2010) The Genome Analysis Toolkit: a MapReduce framework for analyzing next-generation DNA sequencing data. Genome Res 9: 1297-1303.

Pandey MK, Wang ML, Qiao L, Feng S, Khera P, Wang H, Tonnis B, Barkley NA, Wang J, Holbrook CC et al (2014) Identification of QTLs associated with oil content and mapping FAD2 genes and their relative contribution to oil quality in peanut (Arachis hypogaea L.). BMC Genet 15: 133

Sarvamangala C, Gowda MVC, Varshney RK (2011) Identification of quantitative trait loci for protein content, oil content and oil quality for groundnut (Arachis hypogaea L.). Field Crops Res 122: 49-59

Shasidhar Y, Vishwakarma MK, Pandey MK, Janila P, Variath MT, Manohar SS, Nigam SN, Guo B, Varshney RK (2017) Molecular mapping of oil content and fatty acids using dense genetic maps in groundnut (Arachis hypogaea L.). Front Plant Sci 8: 794

Wilson JN, Chopra R, Baring MR, Selvaraj MG, Simpson CE, Chagoya J, Burow MD (2017) Advanced backcross quantitative trait loci (QTL) analysis of oil concentration and oil quality traits in peanut (Arachis hypogaea L.). Tropical Plant Biology 10: 1-17
